# A Conserved BDNF, Glutamate- and GABA-Enriched Gene Module Related to Human Depression Identified by Coexpression Meta-Analysis and DNA Variant Genome-Wide Association Studies

**DOI:** 10.1371/journal.pone.0090980

**Published:** 2014-03-07

**Authors:** Lun-Ching Chang, Stephane Jamain, Chien-Wei Lin, Dan Rujescu, George C. Tseng, Etienne Sibille

**Affiliations:** 1 Department of Biostatistics, University of Pittsburgh, Pittsburgh, Pennsylvania, United States of America; 2 Inserm U955, Psychiatrie Génétique, Créteil, France; 3 Université Paris Est, Créteil, France; 4 Fondation FondaMental, Créteil, France; 5 Department of Psychiatry, University of Halle, Halle, Germany; 6 Department of Human Genetics, University of Pittsburgh, Pittsburgh, Pennsylvania, United States of America; 7 Department of Psychiatry, Center For Neuroscience, University of Pittsburgh, Pittsburgh, Pennsylvania, United States of America; Wayne State University, United States of America

## Abstract

Large scale gene expression (transcriptome) analysis and genome-wide association studies (GWAS) for single nucleotide polymorphisms have generated a considerable amount of gene- and disease-related information, but heterogeneity and various sources of noise have limited the discovery of disease mechanisms. As systematic dataset integration is becoming essential, we developed methods and performed meta-clustering of gene coexpression links in 11 transcriptome studies from postmortem brains of human subjects with major depressive disorder (MDD) and non-psychiatric control subjects. We next sought enrichment in the top 50 meta-analyzed coexpression modules for genes otherwise identified by GWAS for various sets of disorders. One coexpression module of 88 genes was consistently and significantly associated with GWAS for MDD, other neuropsychiatric disorders and brain functions, and for medical illnesses with elevated clinical risk of depression, but not for other diseases. In support of the superior discriminative power of this novel approach, we observed no significant enrichment for GWAS-related genes in coexpression modules extracted from single studies or in meta-modules using gene expression data from non-psychiatric control subjects. Genes in the identified module encode proteins implicated in neuronal signaling and structure, including glutamate metabotropic receptors (GRM1, GRM7), GABA receptors (GABRA2, GABRA4), and neurotrophic and development-related proteins [BDNF, reelin (RELN), Ephrin receptors (EPHA3, EPHA5)]. These results are consistent with the current understanding of molecular mechanisms of MDD and provide a set of putative interacting molecular partners, potentially reflecting components of a functional module across cells and biological pathways that are synchronously recruited in MDD, other brain disorders and MDD-related illnesses. Collectively, this study demonstrates the importance of integrating transcriptome data, gene coexpression modules and GWAS results for providing novel and complementary approaches to investigate the molecular pathology of MDD and other complex brain disorders.

## Introduction

Major depressive disorder (MDD) is a common psychiatric disease with an estimated prevalence of 5.3% for a 12- month period and 13.2% for a lifetime disorder [1], a high rate of recurrence [2], a higher prevalence in women [3], and a heritability of 37% (95% CI = 31%–42%) [4]. Transcriptome (the set of all expressed genes in a tissue sample) and genome-wide association studies (GWAS) have separately provided clues to mechanisms of MDD, although not to the anticipated extent. Transcriptome studies mostly focus on changes in gene expression in disease states (altered expression), but also provide unique opportunities for assessing the less-investigated changes in the coordinated function of multiple genes (altered coexpression) [5]. GWAS seek to identify genetic markers for diseases, and have generated some findings in MDD [6], [7], [8], [9], [10], but overall results from GWAS meta-analyses have been disappointing [11], [12], potentially due to the complexity of the disease and heterogeneity of patient cohorts. GWAS and transcriptome studies are highly complementary in that they provide unbiased and large scale investigation of DNA structural [single nucleotide polymorphisms (SNP) and other variants] and functional (RNA expression) changes across conditions, although these two approaches are only beginning to be integrated [13], [14], [15], [16], [17].

Gene arrays allow for the unbiased quantification of expression (mRNA transcript levels) for 10,000 to 20,000 genes simultaneously. Since gene transcript levels represent the integrated output of many regulatory pathways, the study of all expressed genes provides an indirect snapshot of cellular function under diverse conditions. For instance, using postmortem brain samples, this approach has implicated dysregulated BDNF, GABA, glutamate and oligodendrocyte functions in MDD [18], [19], [20], [21], [22]. However, current studies are still few, were performed in heterogeneous cohorts, and utilized early and rudimentary versions of gene arrays. Moreover, gene array studies are subject to similar limitations as early GWA studies, in that large number of genes are tested in few subjects (n = 10–100). Typical analyses identify 1–10% of genes affected in the illness (differentially expressed genes), are characterized by high rates of false discovery, and may be confounded by numerous clinical (drug exposure, subtypes, duration, etc.), demographic (age, sex, race), technical parameters (RNA integrity, brain pH, postmortem interval for brain collection), or other potential co-segregating factors of unknown origin (See [13] for discussion). Conditions of postmortem brain collection also preclude the reliable identification of acute state-dependent gene changes, but are appropriate for investigating stable long-term disease-related homeostatic adaptations.

Gene coexpression studies offer complementary perspectives on gene changes in the context of transcriptome studies. Here, two genes are defined as coexpressed in a dataset if their patterns of expression are correlated across samples. Coexpression reflects possible shared function between genes, and may arise through multiple biological pathways including cellular coexpression and common regulatory pathways (e.g., hormone signaling, transcription factors) [23], [24]. Hence, coexpression links have been used to build gene networks, and to identify communities, or modules, of genes with shared functions [25], [26]. Notably, by incorporating multiple interactions among a large number of genes, the study of gene coexpression networks provides an approach to tackle the complexity of biological changes occurring in complex polygenic disorders [24]. See [5] for a general review.

Concepts and methods for integrating functional (transcriptome) and structural (DNA polymorphism GWA) studies of the molecular bases of complex neuropsychiatric disorders such as MDD need to be developed to harness the potential of systematic large-scale molecular and genetic investigations of the brain. Here, our central hypothesis states that stable brain co-regulation modules identified through meta-analysis of multiple transcriptome studies may overlap with sets of genes and associated SNPs related to MDD. Based on the continuum of pathological changes between MDD and other brain disorders [27] and co-morbidity with selected medical illnesses including cardiovascular diseases and metabolic syndrome [28], [29], we also predicted that MDD coexpression modules may be enriched in genes identified by GWAS for other psychiatric and brain disorders and potentially for medical illnesses related to depression, together identifying functionally-coherent gene sets implicated in MDD-related disease processes.

## Materials and Methods

**Figure 1:**
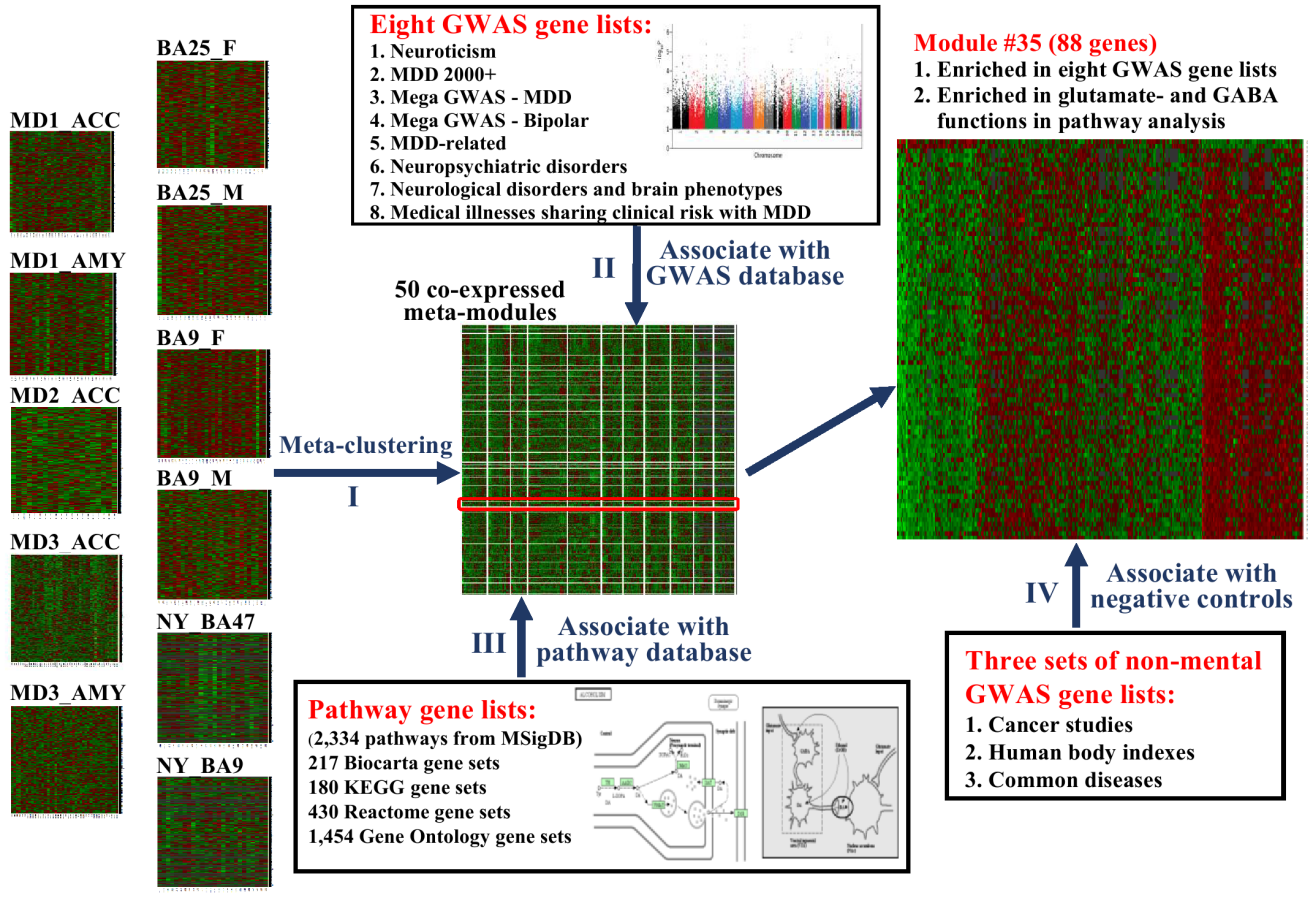
Overall analytical strategy. In step I, 50 co-regulation modules were generated using meta-clustering of gene clusters identified by the “penalized K-medoids” method across 11 transcriptome MDD and matched controls studies. In step II, modules enriched from most of the selected GWAS studies related to MDD, neuropsychiatric disorder and traits, including systemic disease linked to psychiatric disorders were identified. In step III, the biological functions represented by genes included in each module were defined by pathway analysis from 2,334 gene sets of MSigDB (www.broadinstitute.org/gsea/msigdb). In step IV, SNPs from the Catalog of GWAS were organized into three categories: cancer GWAS, human body indices GWAS and GWAS for common diseases and medial illnesses unrelated to MDD or other brain function. Three additional categories were defined as non-MDD-related negative control gene sets. (Note: In order to increase the performance of the heatmap in module #35, we first performed the hierarchical clustering with “complete” agglomeration method to aggregated samples with similar expression among all 88 genes, and the genes were sorted by the correlation from high to low of selected genes in the top.).

**Figure 1** illustrates the meta-clustering and validation methods of the approach. In step I, we identified 50 robust co-regulation modules in human brains by combining 11 transcriptome datasets collected from several brain regions in different cohorts of subjects with MDD and non-affected comparison subjects. Steps II and III were performed to identify MDD-related gene modules, and exclude other gene modules linked to biological functions not related to MDD. In step II, we collected different sets of genes located nearby SNPs identified by GWAS for MDD, neuropsychiatric disorders, related traits, and for systemic diseases often associated with psychiatric disorders, and performed gene set analysis to identify MDD-related gene modules. In step III, we performed functional annotations of gene module members by using 2,334 gene sets collected from MSigDB (http://www.broadinstitute.org/gsea/msigdb/). We also organized genes identified by SNPs in published GWAS into three categories (cancer studies, human body indices and unrelated diseases) and treated them as a non-MDD-related negative control gene sets in step IV.

### Transcriptome Data Sets

Eleven MDD microarray datasets generated in our lab were used here. Cohorts and brain areas investigated are listed in **Table 1** and details were provided in [30], [31]. Among these studies, six used Affymetrix Human Genome U133 Plus 2.0 platforms (Affymetrix Inc., Santa Clara, CA), two used Affymetrix Human Genome U133A platforms, and the remaining three used Human HT-12 arrays from Illumina (Illumina Inc, San Diego, CA). **Figure S1** provides a diagram and results of the transcriptome dataset preprocessing procedures. Data has been deposited to the NCBI Geo database with accession numbers: GSE54562, GSE54563, GSE54564, GSE54565, GSE54566, GSE54567, GSE54568, GSE54570, GSE54571, GSE54572 and GSE54575.

**Table 1:** Description of cohorts in 11 MDD microarray platforms.

Cohort	Region	Code	Platform	# of probe sets	# of genes	# of subjects
1	ACC	MD1_ACC	Affymetrix Human Genome U133 Plus 2.0	40,610	19,466	32
2	AMY	MD1_AMY	Affymetrix Human Genome U133 Plus 2.0	40,610	19,621	28
3	ACC	MD2_ACC	Illumina HumanHT –12 (v3)	48,803	25,159	20
4	ACC	MD3_ACC	Illumina HumanHT –12 (v3)	48,803	25,159	50
5	AMY	MD3_AMY	Illumina HumanHT –12 (v3)	48,803	25,159	42
6	ACC	BA25_F	Affymetrix Human Genome U133 Plus 2.0	53,596	19,572	26
7	ACC	BA25_M	Affymetrix Human Genome U133 Plus 2.0	53,596	19,572	26
8	DLPFC	BA9_F	Affymetrix Human Genome U133 Plus 2.0	53,596	19,572	32
9	DLPFC	BA9_M	Affymetrix Human Genome U133 Plus 2.0	53,596	19,572	28
10	OFC	NY_BA47	Affymetrix Human Genome U133A	20,338	12,703	24
11	DLPFC	NY_BA9	Affymetrix Human Genome U133A	20,338	12,703	26

ACC, anterior cingulate cortex; AMY, amygdala; DLPFC, dorsolateral prefrontal cortex, OFC, orbital ventral prefrontal cortex.

For gene matching across studies, when multiple probes or probe sets match to one gene symbol, we choose the probe set with the largest variation (largest interquartile range; IQR) to represent the gene [32]. See below and Figure S3 for probe overlap assessment. For preprocessing, data were log-transformed (base 2). Non-expressed (small mean intensity) and non-informative (small standard deviation) genes were filtered out. To perform such filtering for 11 studies simultaneously, we calculated the ranks of row means and row standard deviations of each gene in each single study. The ranks were summed up across 11 studies and used as criteria to filter out non-expressed and non-informative genes. Note that ideally we should map the probes across platforms to large overlapped locations so we make sure they measure the same signal. There are, however, several reasons that doing so may not be possible or optimal. First, Affymetrix probesets are designed with combination of multiple short probes and Illumina arrays use a single and longer probe. As a result, Affymetrix probes have large “target regions” (044–728 KB, 95% coverage of the 88 genes of module #35 we investigated in Figure S3) which are covered by multiple short probes, while Illumina’s probe is only around 50 bp. Secondly, many other factors affect signal detection efficiency, including exact probe sequence, integration of multiple probeset in Affymetrix arrays, hybridization efficiency, GC content, cross hybridization, etc. As a whole these differences can affect the consistency of the results and potentially decrease the final signal. For the purpose of running the meta-analysis (as opposed to single study analysis) it has been recommended to use the probe set with the largest IQR to represent a gene symbol [32]. We want to point out that if the IQR probe matching procedure had introduced large errors, the meta-analyzed modules would not have been detected by chance.

### Meta-clustering of Transcriptomic Data to Construct Co-expression Gene Modules

The 11 transcriptome studies were combined to construct co-expression gene modules using a meta-clustering technique described below. We denoted by *X_gsk_* the gene expression intensity of gene *g*, sample *s* and study *k*, and *X_gk_* = (*X_g1k_*,…, *X_gSk_*) the vector of gene expression intensities of gene *g* and study *k*. We defined the dissimilarity measure between gene *i* and gene *j* for a given study *k* as 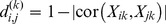, where cor(*X_ik_*, *X_jk_*) is the Pearson correlation of the two gene vectors. To combine the dissimilarity information of the *K = *11 studies, we took the mean of meta-dissimilarity measure between gene *i* and gene *j* as 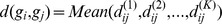. Given the meta-dissimilarity measure, the “Penalized K-medoids” clustering algorithm was then applied to construct co-expression gene modules [33]. The target function to be minimized by Penalized K-medoids is shown below
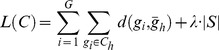
where the clustering result 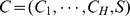 contains *H* non-overlapping gene clusters (i.e. *H* gene modules 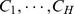) and a set of scattered genes *S* that cannot be clustered into any of the tight gene modules, 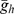 denotes the medoid gene of cluster *h* such that its average dissimilarity to all other genes in the cluster is minimal, 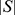 is the size of the scattered gene set *S* and *λ* is a tuning parameter controlling tightness of detected gene modules and the number of scattered genes discarded to *S*. The first term of the target function *L*(*C*) calculates the total sum of within-cluster dispersion and is essentially the K-medoids algorithm (an extended form of K-means using arbitrary non-Euclidean dissimilarity measure). The second penalty term allows scattered genes not to be clustered into any gene module. For example, if the distances of a gene *g_i_* to all cluster medoids are greater than *λ*, minimizing *L*(*C*) will assign the gene into the scattered gene set *S*, instead of into any gene cluster. Intuitively, smaller *λ* generates tighter clusters and allow more genes into scattered gene set *S*. The rationale for the choice of this approach was based on finding in the literature, where comparative studies show that many genes are not tightly co-expressed with any gene clusters and methods that allow scattered gene assignment generates tighter gene modules that are biologically more informative [34].

### Parameter Selection and Evaluation of Meta-clustering

We tested different parameter settings of *H* = 50 or 100 modules, and *λ* such that *β* = 0%, 25% or 50% of genes are left to scattered gene set *S*. In all performance of the 2×3 = 6 combinations for the meta-clustering method, a biological validation was performed using biological pathway information. We searched ten keywords (“GABA”, “Insulin”, “Diabetes”, “Immune”, “Thyroid”, “Estrogen”, “Depression”, “Alzheimer”, “Parkinson” and “Huntington”) in MSigDB and finally obtained 98 MDD-related pathways. In each clustering result, Fisher’s exact test was applied to each module to correlate with each of the 98 MDD-related pathways and eight GWAS gene lists and the p-values were generated. Wilcoxon signed rank test was used to compare any pair of clustering results (from different parameter setting) so that the best parameter setting could be determined.

### Evaluation of Robustness and Stability of Meta-clustering Method

To evaluate the robustness of the meta-clustering results, we used the Adjusted Rand Index (ARI) as a measurement of consistency between two clustering results [35]. Specifically, ARI calculates the proportion of concordant gene pairs across two clustering results (i.e. two genes are clustered together in both clustering results or not clustered together in both) among all possible gene pairs and the index is standardized between 0 and 1, where 0 reflects expected similarity measure of two random clustering and 1 reflects similarity measure between two identical clustering. We randomly selected a subset of studies (n = 8, 9 or 10) from 11 MDD studies and calculated the ARI to assess the similarity of the obtained modules compared to those obtained using the 11 MDD studies. The procedure was repeated 100 times and the average ARI was calculated. For the stability of meta-clustering method, the mean and standard deviation of ARIs were obtained by bootstrapping method [36] (sampled with replacement to obtain the same number of samples for each single study), where the 11 MDD studies were bootstrapped 100 times.

### Genome-wide Association Studies (GWAS)-related Gene Categories

Eight neuropsychiatry-related candidate gene lists and three gene lists from presumably unrelated disorders or traits were identified from relevant GWAS. Individual genes were identified by the presence of GWAS significant SNPs within a given nucleotide distance from the coding region of that gene (UCSC hg18 with build 36.3 was used for all GWAS).

The first gene list was obtained from a published GWAS for neuroticism [37]. Neuroticism is a personality trait that reflects a tendency toward negative mood states, and that is linked to several internalizing psychiatric conditions. That GWAS involved 1,227 healthy individuals with self-report of no diagnosis or treatment for schizophrenia, schizoaffective disorder or bipolar disorder and personality measures of neuroticism. Genotyped data were generated from Affymetrix GeneChip Human Mapping 500 K using BRLMM algorithm. 449 SNPs were selected by p-value less than 0.001, and 155 genes were identified to have contained one or more selected SNPs in the 10 kilobases (kb) up- and down-stream extension of the coding regions.
The second gene list was obtained from the MDD 2000+ project that included a meta-analysis of MDD studies with 2,431 MDD cases and 3,673 controls [38]. Similarly, 532 SNPs with p-value less than 0.001 were mapped to gene coding regions (including 10 kb upstream and downstream regions) and 159 genes were identified.
The third gene list was obtained from a mega-analysis of GWAS for MDD [11]. The associated 202 SNPs’ p-values were less than 10^−5^ and 52 genes were identified using the University of California Santa Cruz Human Genome Browser, hg18 assembly (UCSC hg18) with build 36.3. Gene symbols from the build version 36.3 in the National Center for Biotechnology Information (NCBI) database were used.
The fourth candidate gene list was obtained from a mega-GWAS of bipolar disease which contained 7,481 patients and 9,250 controls [39]. 6,887 SNPs were identified when p-value less than 0.001. By mapping the SNPs to gene coding region using SNPnexus software (http://snp-nexus.org/), 602 genes were obtained.
For the fifth to eighth gene lists, we interrogated the Catalog of Published Genome-Wide Association Studies [40] (http://www.genome.gov/gwastudies/). The database (as of 01/31/13; time of the latest data analysis update) contained 10,183 entries of disease- or trait-associated SNPs with p-values smaller than 10^−5^ in 1,491 GWAS studies. We manually regrouped the disorders and traits into 4 categories: (1) all MDD-related studies, (2) all neuropsychiatric disorder studies, (3) all neurological disorder and brain phenotypes studies, (4) all medical illnesses sharing increased risk with MDD. Note that the genes in the list #3 were included in the list #2, and genes in the list #2 were included in the list #1, which is the larger category (see more detail list in **Table S1**). Lists #4 is independent and non-overlapping with others. The associated four gene lists were then compiled, and genes were uniquely included when the mapped SNP was within the gene region including a 100 kb upstream and downstream.
As negative controls, we identified in the catalog of published GWAS three gene sets presumably not related to psychiatric diseases: (a) 65 publications (270 genes) of cancer GWAS studies; (b) 42 publications (459 genes) of human body indices GWAS studies (HBI: genetic phenotypes for human, for example: height, weight, eye color, etc.); and (c) 33 publications (187 genes) of GWAS studies for common disease traits not related to brain function or major mental illnesses (**Table S2**).

### Meta-analysis to Aggregate Evidence of Association of each Module with the GWAS Gene Lists

We performed Fisher’s exact test to examine the significance of the association of genes within each coexpression module with individual GWAS-derived gene lists, using the 10,000 genes evaluated in transcriptome meta-analysis (**Figure S1**) as background. To assess statistical significance of association of each identified module from meta-clustering method, we applied the Stouffer’s method to combine the *p*-values obtained from Fisher’s exact test of the association between gene modules and eight GWAS gene sets. The Stouffer’s statistics 
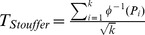
 where *φ* is the cumulative distribution function of a standard normal distribution [41]. The p-values were assessed for each of the 50 modules from non-parametric permutation analysis by randomly selecting the same number of genes from the whole genome without replacement (using genome background 10,000 genes) for each of the 50 modules and the analysis is repeated for 500 times.

### Pathway Analysis and Enrichment Analysis of GWAS Gene Lists

For biological association, 2,334 annotated pathways (gene sets) were obtained from MSigDB (www.broadinstitute.org/gsea/msigdb/), which consists of 880 canonical pathways (217 Biocarta gene sets, 180 KEGG gene sets, 430 Reactome gene sets and 53 other gene sets) and 1,454 pathways from Gene Ontology (GO). For each of the gene module, gene set (pathway) analysis was performed for the 2,334 pathways and 11 GWAS gene lists (including 3 negative controls). Fisher’s exact test was performed to assess the biological association between gene modules and given gene sets. To account for multiple comparisons, Benjamini and Hochberg procedure was used to control the false discovery rate (FDR) [42].

## Results

### Data Preprocessing and Parameter Determination

16,443 genes were retained after gene matching across the 11 studies. Cohorts 10 and 11 were from older platforms with fewer probesets representing only 12,703 genes (**Figure S1**). In order to minimize the loss of information from gene matching, we allowed 20% missing values during matching, i.e., we kept genes with at least 9 existing measurements out of 11 studies. 13,500 genes were retained after filtering out lower sum rankings of median row means, and 10,000 genes after further filtering out lower sum rankings of median row standard deviations. We then tested different parameter settings for the number of modules (*H* = 50 or 100), and genes (tuned the *λ* values for controlling tightness of detected gene modules and the number of scattered genes set) for *β* = 0%, 25% or 50% of genes left out of the gene set *S*. In all tests of the Penalized K-medoids meta-clustering method (2×3 = 6 combinations), we performed a validation by biological pathway information content. For all clustering results, Fisher’s exact test was applied to each module to correlate with each of the 98 MDD pathways and eight GWAS gene lists described in the methods, and p-values were generated. The Wilcoxon signed rank test was used to compare any pair of clustering results (from different parameter settings) so that the best parameter setting could be determined. The result shows that there was no significant difference (by Wilcoxon signed rank test) between *H* = 50 and *H* = 100 clusters except *β* = 0% (i.e., keep all genes), and the minimum p-value of gene set analysis in *H* = 50 was always lower than that in *H* = 100 in *β* = 25% and *β* = 50%. It is reasonable to set the noise level in clustering method because noise will increase if we combined more studies. We chose *H* = 50 because the mean of the –log10(p) in 50 modules (3.2793) was higher than 100 modules (3.0224) in *β* = 25%, and the mean of the –log10(p) in 50 modules (3.1896) was higher than 100 modules (3.0588) in *β* = 50%. 50 modules also provide adequate number and sizes of gene modules for the purpose of further analyses. Given *H* = 50, we compared the performance with different choices of *β*. *β* = 25% performed better than *β* = 0% (p = 0.0004 using Wilcoxon signed rank test), and there was no significant difference between *β* = 25% and *β* = 50% (p = 0.0856). Finally, we selected *H* = 50 and tuning parameter *λ* such that *β* = 25% genes are left to scattered gene set *S* throughout this paper (**Table S3**).

### Construction of 50 Meta-modules from 11 MDD Studies

Using the parameters determined above, we performed a meta-analysis of module gene membership to identify the top 50 meta-analyzed coexpression modules across 11 MDD transcriptome studies. A total of 10,000 genes were clustered using the Penalized K-Medoid method. 7,797 genes were clustered into *K* = 50 modules and 2,203 genes (*β* = ∼25%) were determined as scattered genes with no coherent expression pattern. We performed subsampling and bootstrap methods to assess the stability of the resulting clusters. Subsets (n = 8, 9 or 10) of the 11 studies were randomly selected and the meta-clustering procedure was similarly applied. The resulting meta-modules were compared with the meta-modules obtained using the 11 MDD studies using adjusted Rand index (ARI = 0.47, 0.52 and 0.63 for n = 8, 9, 10). We also generated bootstrapped samples in each study and repeated the meta-clustering procedures. Comparison of meta-modules generated from bootstrapped samples with original samples generated an average ARI = 0.45 (standard deviation 0.025) in 100 repeated bootstrapping simulations. In the literature, an ARI of ∼0.5 is interpreted as reproducible clustering result [34], hence demonstrating good stability under data perturbation (subsampling and bootstrapping) for the 50 meta-modules obtained by combining 11 studies.

### Association of Meta-modules with Eleven GWAS-determined Gene Lists

**Figure 2:**
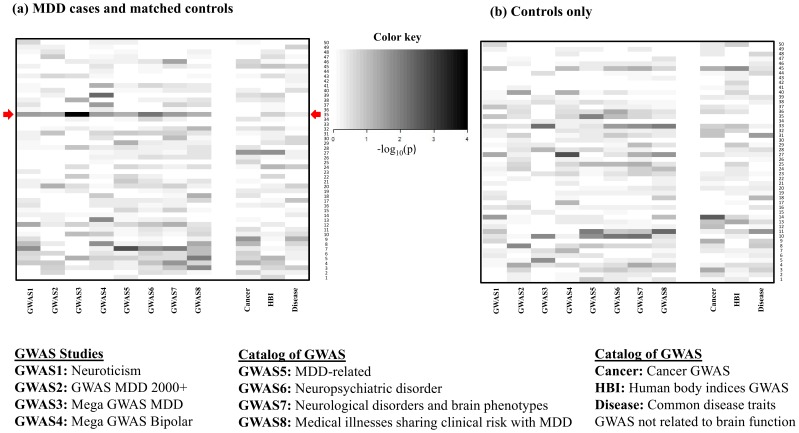
Consistent association of genes in module #35 with MDD-related gene categories. (**a**) Heatmap of log_10_-transformed p-values from Fisher’s exact test for 50 modules obtained from MDD cases and matched controls and 8 MDD related GWAS and 3 negative controls. (**b**) Heatmap of log_10_-transformed p-values from Fisher’s exact test for 50 modules obtained from controls and 8 MDD related GWAS and 3 negative controls. The green rectangle identifies module #35.

We examined association of the 50 meta-modules with the eight GWAS gene lists using Fisher’s exact test. The results are shown in **Table S4**. Module #35 is found to have significant associations (p<0.05) with the six psychiatric disorder related GWAS gene sets (p = 0.03 for the neuroticism GWAS gene set; p = 0.03 for MDD 2000+ project; p = 0.0001 for Mega-GWAS MDD; p = 0.03 for Mega-GWAS of bipolar disorder; p = 0.008 for the catalog of GWAS studies of neuropsychiatric disorder; p = 0.03 for the catalog of GWAS studies of neurological disorders and brain phenotypes) and two studies with borderline p-values (p = 0.05 for the catalog of MDD-related GWAS studies; p = 0.05 for the catalog of GWAS studies of Medical illnesses sharing clinical risk with MDD). We combined the p-values of the eight psychiatric disorder related GWAS gene sets by Stouffer meta-analysis method. The p-value of module #35 is 4e^−05^ after the permutation test (25,000 resamples). In contrast, there was no association with cancer (p = 1.00), human body indices (p = 0.18) and other control diseases (p = 0.46) GWAS gene sets. **Figure 2** **(a)** shows the heatmaps of log-transformed p-values from enrichment analysis for the 50 modules obtained from MDD cases and controls combined analysis. It shows that module #35 (highlighted in green) from the combined cases and controls analysis is enriched in genes contained in six MDD-related GWAS gene sets, but not enriched in the three negative control GWAS gene sets. None of the other 49 modules showed such consistent pattern.

During the review process, a new GWAS meta-analysis for schizophrenia was published by the Psychiatric Genomics Consortium (PGC) using 1,000 genome Project imputation [43]. Accordingly, we independently examined the reported 52,509 SNPs spanning 2,507 genes under the p-value threshold of 10^−3^ (Table S9). Module #35 was significantly enriched in genes associated with this new study (p = 0.0013).

### Pathway Analysis of Meta-module #35

**Table S6** lists detailed information of the 88 genes in module #35 and their overlap with the eight GWAS gene lists. Many GWAS-hit genes were related to synaptic function, signal transduction, and neuronal development and morphogenesis (**Table 2**). Of specific interest, and consistent with current hypotheses for the molecular pathology of MDD, was the inclusion of brain-derived neurotrophic factor (BDNF) and other factors implicated in development and maintenance of cell circuits (Ephrin receptors EPHA3 and EPHA 5; Netrin G1 (NTNG1); SLITRK3 and SLITRK5), of GABA-related genes (GABBR2, GABRA4 and CALB1), glutamate receptors (GRM1 and GRM7) and other signaling neuropeptides previously implicated in mechanisms of psychiatric disorders [reelin (RLN) and gastrin-releasing peptide (GRP)] (**Table 2**). Results from a pathway enrichment analysis confirmed the role of genes in module #35 in overall signaling mechanisms (**Table 3**). Together, these results suggest that module #35 may include multiple components of functionally-relevant local cell circuits.

**Table 2:** Functional groups of 88 gene in module #35**.**

Functional groups	Gene Symbols
Transmembrane cellular localization	*CLSTN2, SYT4, LRRC8B, GPR6, TMEM158*
	*ST8SIA3, GABBR2, NRN1, ST6GALNAC5*
	*GLT8D2, MPPE1, GNPTAB, PVRL3, SLC35B4*
	*SLC35F3, KCNG3, SLC30A9, PTGER4, CYP46A1*
	*GABRA4, UST, LOC646627, NTNG1, TMEM200A*
	*TMEM70, RFTN1, GRM1, TMEM132D, KCNV1*
	*EPHA3, CDH12, EPHA5, BEAN, SLITRK3*
	*FREM3, GRM7, CD82, SLITRK5, VLDLR*
Neuronal development and morphogenesis	*BDNF, SLITRK3, RPGRIP1L, MAEL, NTNG1,*
	*RELN, LAMB1, SLITRK5, MYCBP2d*
GABA and glutamate	*GRM1, GRM7, GABBR2, GABRA4*
Cell adhesion	*PPFIA2, CDH12, FREM3, CLSTN2, PVRL3, RELN*
	*LAMB1*
Transcription regulation	*EGR3, DACH1, HDAC9, ATOH7, SLC30A9*
	*ATF7IP2, ZNF436, MYCBP2*

Annotations are based on Gene Ontology. See Table 3 for a separate analysis of pathway enrichment.

**Table 3:** Top 15 enriched pathways in module #35.

Pathways	P-values
METABOTROPIC_GLUTAMATE_GABA_B_LIKE_RECEPTOR_ACTIVITY	0.0003
REACTOME_CLASS_C3_METABOTROPIC_GLUTAMATE_PHEROMONE_RECEPTORS	0.0005
G_PROTEIN_SIGNALING_COUPLED_TO_CAMP_NUCLEOTIDE_SECOND_MESSENGER	0.002
CAMP_MEDIATED_SIGNALING	0.002
GLUTAMATE_RECEPTOR_ACTIVITY	0.003
G_PROTEIN_COUPLED_RECEPTOR_PROTEIN_SIGNALING_PATHWAY	0.003
G_PROTEIN_SIGNALING_COUPLED_TO_CYCLIC_NUCLEOTIDE_SECOND_MESSENGER	0.008
CYCLIC_NUCLEOTIDE_MEDIATED_SIGNALING	0.01
NEUROPEPTIDE_HORMONE_ACTIVITY	0.015
REACTOME_GPCR_LIGAND_BINDING	0.02
KEGG_NEUROACTIVE_LIGAND_RECEPTOR_INTERACTION	0.03
G_PROTEIN_COUPLED_RECEPTOR_ACTIVITY	0.03
SECOND_MESSENGER_MEDIATED_SIGNALING	0.04
HORMONE_ACTIVITY	0.04
REACTOME_EICOSANOID_LIGAND_BINDING_RECEPTORS	0.04

### Control Studies

**Figure 3:**
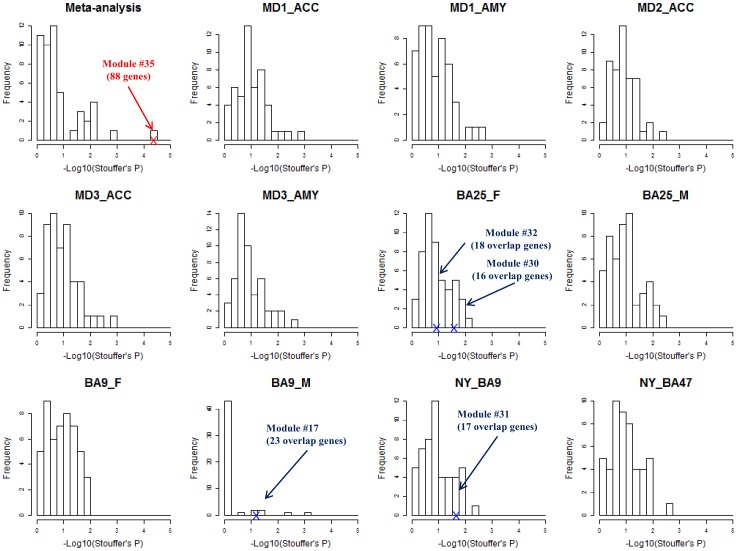
Histograms of the –log_10_(p) of the Stouffer statistic from 50 modules of meta-analysis of 11 MDD studies and each single study. Module #35 with 88 genes (red arrow and double-cross) have largest –log_10_ transformed p-value of Stouffer’s statistic 4.4. The other four blue arrows and double crosses indicated that these four modules in all single studies have more than 14 (15% of the 88 genes in module #35) overlapped with module #35. See detailed description in text.

To demonstrate the improvement of meta-clustering versus single study clustering, we compared the histograms of p-values obtained under those different conditions. In **Figure 3**, the histogram of the minus log-transformed p-values of the Stouffer statistic was first plotted for the 50 meta-modules obtained from the case and control combined analysis. Module #35 with 88 genes is shown to have an aggregated minus log-transformed p-value at 4.4 (i.e. p = 4e-05). We then applied the penalized K-medoid method with the same parameter setting (K = 50 clusters and 25% of scattered genes) for each single study. The 11 single study histograms of Stouffer p-values showed overall much weaker statistical significance than for module #35. Particularly, none of the 550 modules from 11 single study cluster analysis was enriched (p-value threshold 0.05) in more than three GWAS results (**Figure 3**). Only four out of the 550 modules had more than 14 genes (∼15% of the 88 genes; indicated by blue arrows in **Figure 3**) that overlapped with module #35. Hence, the meta-clustering approach efficiently combined weak signals in single studies to identify a stable and biologically more meaningful gene module. In other words, module #35 would not have been discovered without combining 11 studies.

We next tested the meta-clustering approach using transcriptomic data from control subjects only (i.e., removing all MDD subjects) from the same 11 studies. Out of the 50 modules generated, no module was enriched in more than two GWAS studies (p-value threshold 0.05) among the eight GWAS results (see **Table S5** and heatmap in **Figure 2** **(b)**), indicating that the inclusion of the MDD cases was necessary for the detection of significant module/GWAS overlap (i.e. module #35). We note that this comparison is not a “proof” of the significance of module #35 since the “control-only” analysis contains only half the sample size of the “cases+controls”. To investigate the impact of the sample size, we randomly sampled half cases and half controls to perform “cases+controls” versus “control-only” comparisons. We meta-analyzed the p-values of the eight enrichment analyses (using Stouffer’s method) in each module and retain the most significant one (i.e. smallest p value) among the 50 modules. The procedure was repeated for 20 times. The result shows that meta-coexpression analysis using case and control samples combined has better detection power to identify modules associated with neuropsychiatric diseases. In **Figure S2**, the red cross shows result of “control-only” and the histogram shows the 20 subsampled “cases+controls”. The result from the full “cases+controls” is also shown for reference (blue cross). We also tested the meta-clustering approach using transcriptomic data from MDD subjects only (i.e., removing all control subjects) from the same 11 studies. Among the 50 modules generated, one module (module #15 with 169 genes) was enriched in six out of the 8 GWAS categories (p<0.05) but notably not in the gene set corresponding to the Mega GWAS MDD (p = 0.29) and to MDD-related studies (p = 0.43) in the catalog of GWAS (**Table S7**). This module only has 3 genes overlapped with the 88 genes (ST8SIA3, GRM7 and MYCBP2) of module #35 extracted from the case and control combined analysis. Pathway analysis of this module indicated an over-representation of signal transduction pathways (**Table S8**). Overall, the statistical significance of results using MDD data only was lower and potentially inconclusive (i.e. at background noise level).

Together these results indicate that combining MDD and control subjects in meta-clustering approaches increased the significance and robustness of the results, as demonstrated by the identification of the tight module of 88 genes with high relevance to current biological knowledge about MDD.

## Discussion

Using methods we developed to identify meta-analyzed coexpression modules across transcriptome datasets, we report the identification of a module consisting of 88 genes that is significantly enriched in genetic variants located nearby genes otherwise associated with major depression and related phenotypes. The finding of a significant intersection of two unbiased large-scale approaches (transcriptome and GWAS) provide robust evidence for the putative recruitment and contribution to molecular and cellular mechanisms of MDD of a biological module that is formed by the identified gene set. This module includes numerous genes encoding proteins implicated in neuronal signaling and structure, including glutamate metabotropic receptors (GRM1, GRM7), GABA-related proteins (GABRA2, GABRA4, CALB1), and neurotrophic and development-related molecules [e.g., BDNF, reelin (RELN), Ephrin receptors (EPHA3, EPHA5)]. These findings are consistent with current hypotheses of molecular mechanisms of MDD, notably with the GABA, glutamate and neurotrophic hypotheses of depression [27], [44], [45], [46], [47]. This biological “internal validation”, combined with control studies showing that these results could not be achieved using single studies (due to weak signal) demonstrates that integrating transcriptome data, gene coexpression modules and GWAS results can provide a novel and powerful framework to improve understanding of MDD and other complex neuropsychiatric disorders. This approach also provided here a set of putative interacting molecular partners, potentially reflecting a core biological module that is recruited and implicated in biological mechanisms of MDD.

The meta-clustering approach in this paper has the following novelty and advantages. (1) *Meta-analysis*: Our result indicated that a meta-analysis of gene clustering to combine multiple transcriptome studies can identify more accurate and robust gene modules, since the same clustering method applied to single studies did not lead to the identification of any significant and/or neuropsychiatry-related module. (2) *Cluster analysis allowing “scattered genes”*: Gene coexpression modules were identified by penalized K-medoid. This clustering technique searches for tight gene modules and allows some genes to be scattered. This means that they are not included in the final set of modules/clusters, unlike other traditional clustering methods, such as hierarchical clustering, K-means or self-organizing maps that force all genes into clusters. In genomic applications, it was shown that allowing scattered genes can improve clustering performance with better biological knowledge discovery [34]. (3) *Integration and validation with external databases*: Integration with rich GWAS and pathway knowledge databases for biological and disease interpretation identified a robust module with 88 genes that is consistent with current knowledge about depression, hence providing some level of “internal control” for the methods. (4) *Case and control combined co-expression analysis*: We showed that the combination of case and control coexpression analysis was necessary to reveal the co-expression perturbation originating from the disease. This is an important observation as coexpression studies rely on subtle differences in expression patterns compared to differential expression between two groups. Hence disease-related coexpression modules could have been predicted to be unique to the disease groups and “diluted” when combined with control data. However, we show that the opposite is true, resulting in increased power in the combined dataset. For technical validation, we have performed the following: First, we fine-tuned the parameters to be used in the final meta-clustering analysis (i.e. number of modules, percentage of allowed scattered genes in penalized K-medoid method) and tested those parameters in three studies using “surrogate” information, i.e. gene families and biological pathways broadly associated with psychiatric disorders (See Methods). Second, subsampling and bootstrap simulation were applied to investigate the stability of the identified gene modules. Third, three non-psychiatric related GWAS gene sets (cancer, human body indexes and disease traits unrelated to mental functions) served as negative controls.

Coexpression links between genes are inferred from microarray expression studies but do not refer to any specific mechanism underlying these correlations. In fact, any mechanism that synchronously regulates transcription of multiple genes may potentially generate coexpression relationships, including biophysical sources (e.g., transcription factors, spatial configuration of chromosomes, mRNA degradation, miRNA or other upstream regulation, histone acetylation and methylation patterns), technical effects (e.g., batch processing, RNA quality), cell biological sources (e.g., cellular admixture of the sampled tissue, brain region), and importantly synchronized activities across cells under homeostatic equilibria corresponding to “control” states, trait conditions, or chronic disease states for instance. Here, results in module #35 identify a set of genes whose products are distributed across cell types, cellular compartments and biological processes (**Tables 2** **–** **3**) that together contribute to various and potentially complementary biological processes, and whose collective function may be related to pathological processes implicated in depression.

The biological content of the identified gene module is notable in that it brings together multiple genes that have been otherwise associated with depression and other neuropsychiatric disorders through multiple studies both in humans and animal models, in addition to the genetic links (i.e., GWAS) that were used here to identify them. Such commonly associated genes include those coding for BDNF, and GABA- and glutamate receptors, for instance [18], [19], [20], [21], [22]. Prior findings often refer to differential expression, e.g. reduced BDNF [22], or reduction in calbindin (CALB1) positive GABA neurons [48]. Here, reports of conserved co-regulated patterns between these genes suggests that changes in the fine-tuning and synchronization of the function of these gene products across cells and pathways may contribute to pathophysiological mechanisms related to brain dysfunction in MDD. The fact that these results implicate genes that are likely to be expressed across cell types or to regulate ensembles of cells (i.e. neurotrophic and neuro-maintenance factors) is consistent with mechanisms expected for polygenic complex disorders. Moreover, the identification of module #35 through overlap with GWAS findings for traits (i.e. neuroticism) and other neuropsychiatric disorders (**Figure 2**) also suggests that those genes may participate in basic cellular functions that are implicated in a continuum of biological states (i.e., from normal to disease brain functions), consistent with a dimensional understanding of biological mechanisms of brain disorders. The fact that borderline significance in gene overlap was also observed for categories of disorders sharing clinical risk with MDD (i.e. cardiovascular diseases, inflammation and metabolic syndrome) suggest that the same gene sets may also contribute to dysfunctions in peripheral organs through pleiotropic functions of common genes, hence providing putative biological links for the clinical and symptom co-morbidity. Follow-up studies of coexpression patterns obtained in datasets across these disorders may be necessary to further investigate these interesting hints.

So while these studies provide insight into the biology of complex disorders, one may reasonably ask how they may contribute to the generation of novel hypotheses and predictions. Two directions are worth mentioning. First, for the purpose of therapeutic development and target identification, the application of graph theory and other network analysis may help identify critical genes within the identified module or upstream factors, as potential mediators of the function of this module in disease state. Preliminary analyses of the network properties of module #35 did not provide clear insight into hub genes or other parameters of interest (data not shown); however these studies may be confounded by circular analyses within the same datasets. Thus, testing these hypotheses in other large-scale disease related datasets are needed to, firstly, refine gene membership into the identified module, in view of the reasonable and significant conservation of module structure across datasets, although not to absolute levels; and, secondly, to identify key network nodes with conserved cross-studies functions, as potential targets to modulate the functional outcome of the identified gene module. Finally, an additional and important outcome of these studies is that they provide a focused set of genes, which can be used for follow-up genetic association studies, hence potentially mitigating the problem of reduced statistical power of large scale genome-wide studies.

There are several limitations to this study. First, there is a bias when selecting gene sets from the catalog of published GWAS results since the targeted markers (SNPs) are updated every six months, and many more SNPs were reported in the past five years when GWAS have achieved greater sample size (including studies with more than 10,000 participants) and detection of markers with very small effect size. However, large sample sizes will also introduce a bias towards false positive markers. A related limitation is that the choice of markers (or gene) was based on fixed and arbitrary thresholds (i.e., p-value and genomic distance). Moreover, we used only a small fraction of the datasets and pre-defined pathways related to psychiatric disease to decide on the number of clusters and sets of scattered genes during the method development phase, so the result of the clustering approaches may still show some instability and may vary based on different numbers of clusters and applied thresholds. Indeed, although we performed extensive validation analyses to select the clustering parameters and increase stability of modules, the 88 genes in module #35 will inevitably vary slightly under additional data perturbation (e.g., when adding additional MDD or related studies). An additional limitation is that generating gene coexpression modules using cluster analyses is known to be sensitive to small data perturbation. To mitigate these effects, we combined multiple studies and concentrated on tight modules by leaving out scattered genes. While this approach increased the power of the meta-clustering method, it also meant combining datasets from different brain regions, hence potentially diluting the effects of local coregulation patterns that may be important for disease mechanisms. The integration of multiple datasets comes at the expense of variable technical platforms, including inclusion of different probesets across array types. We investigated this potential issue and showed considerable overlap in genomic region targeted by the various probes for a same gene (**Figure S3**), hence lowering the potential impact of this array differences. So these results should be considered proof-of-concept, rather than experimentally and biologically optimized. Finally, it is important to note that changes in gene coexpression are difficult to confirm by independent measures. Indeed coexpression links rely on large sample size and we previously showed that the sample-to-sample variability in array-based measures of expression is typically lower than the variability obtained using alternate measures such as quantitative PCR [24], so the ultimate test of the added value of these meta-coexpression studies will need to come from additional independent studies. Nonetheless, this study allowed the identification of a focused set of genes for use in future genetic association studies, and together demonstrates the importance of integrating transcriptome data, gene coexpression modules and GWAS results, paving the way for novel and complementary approaches to investigate the molecular pathology of MDD and other complex brain disorders.

## Supporting Information

Figure S1 **Diagram of pre-processing procedure of 11 MDD transcriptiome data sets.** Number of samples and number of matched genes in each single (MDD) study. In matching step, we allowed 20% missing studies, then 16,443 genes were identically matched among 11 studies. 13,500 genes were kept by filtering out lower sum ranks of median row means; 10,000 genes were kept by filtering out lower sum ranks of median row standard deviations.
(TIFF)

Figure S2 **Histogram of minimum log_10_-transformed p values from Stouffer’s statistics for 50 modules obtained from randomly selected MDD cases and matched controls into half for 20 times.** Red cross represents the minimum log_10_-transformed p value for controls only study and blue cross represents the minimum log_10_-transformed p value for cases plus controls study.
(TIFF)

Figure S3 **Sequence target overlap between Affymetrix and Illumina array probesets.** We have systematically mapped the respective probes that were chosen by our approach and used genes in module #35 to specifically look at overlap in targeted regions. As shown in the individual graphs below, there is overlap in regions for 94% of the genes, indicating that for a few exceptions the same transcript region is used. A histogram represents a chromosomal area of a target sequence in either affymetrix or illumina platform. Wider histogram means the target sequence span over DNA sequence more widely. The height of each histogram shows the number of studies use that specific probe. See main text for additional information.
(TIFF)

Table S1 **Categories of the GWAS.**
(XLSX)

Table S2 **Disease traits of negative controls from catalog of GWAS.**
(XLSX)

Table S3 **Wilcoxon signed rank test of parameter determination.**
(XLSX)

Table S4 **Meta modules and GWAS gene lists (cases and controls).**
(XLSX)

Table S5 **Meta modules and GWAS gene lists (controls only).**
(XLSX)

Table S6 **GWAS-hit genes in module #35.**
(XLSX)

Table S7 **Meta modules and GWAS gene lists (cases only).**
(XLSX)

Table S8 **Pathway analysis.**
(XLSX)

Table S9 **Meta modules and PGC schizophrenia.**
(XLSX)
