# Supplementary figures and images for: A Conserved BDNF, Glutamate- and GABA-Enriched Gene Module Related to Human Depression Identified by Coexpression Meta-Analysis and DNA Variant Genome-Wide Association Studies
Source: PLoS One. 2014 Mar 7;9(3):e90980. doi: 10.1371/journal.pone.0090980 (PMC3946570; doi:10.1371/journal.pone.0090980)

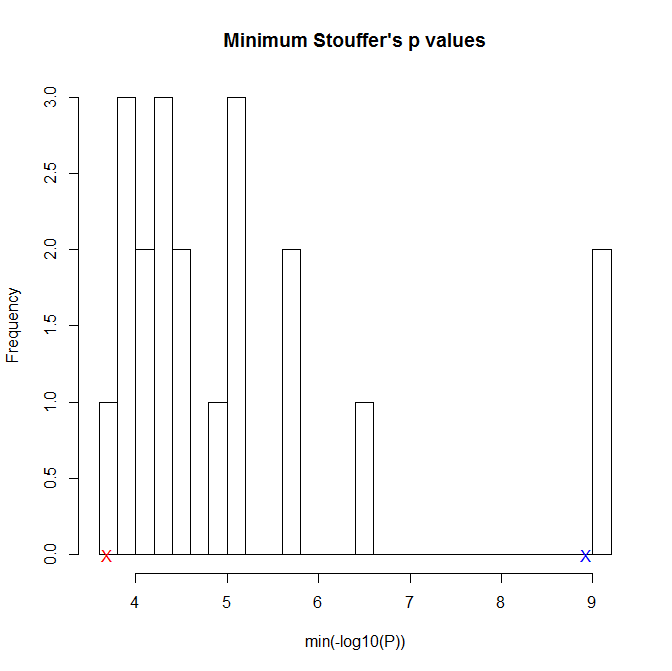

Supplement: Figure S2 — Histogram of minimum log10-transformed p values from Stouffer’s statistics for 50 modules obtained from randomly selected MDD cases and matched controls into half for 20 times. Red cross represents the minimum log10-transformed p value for controls only study and blue cross represents the minimum log10-transformed p value for cases plus controls study. (TIFF) [file pone.0090980.s002.tif]

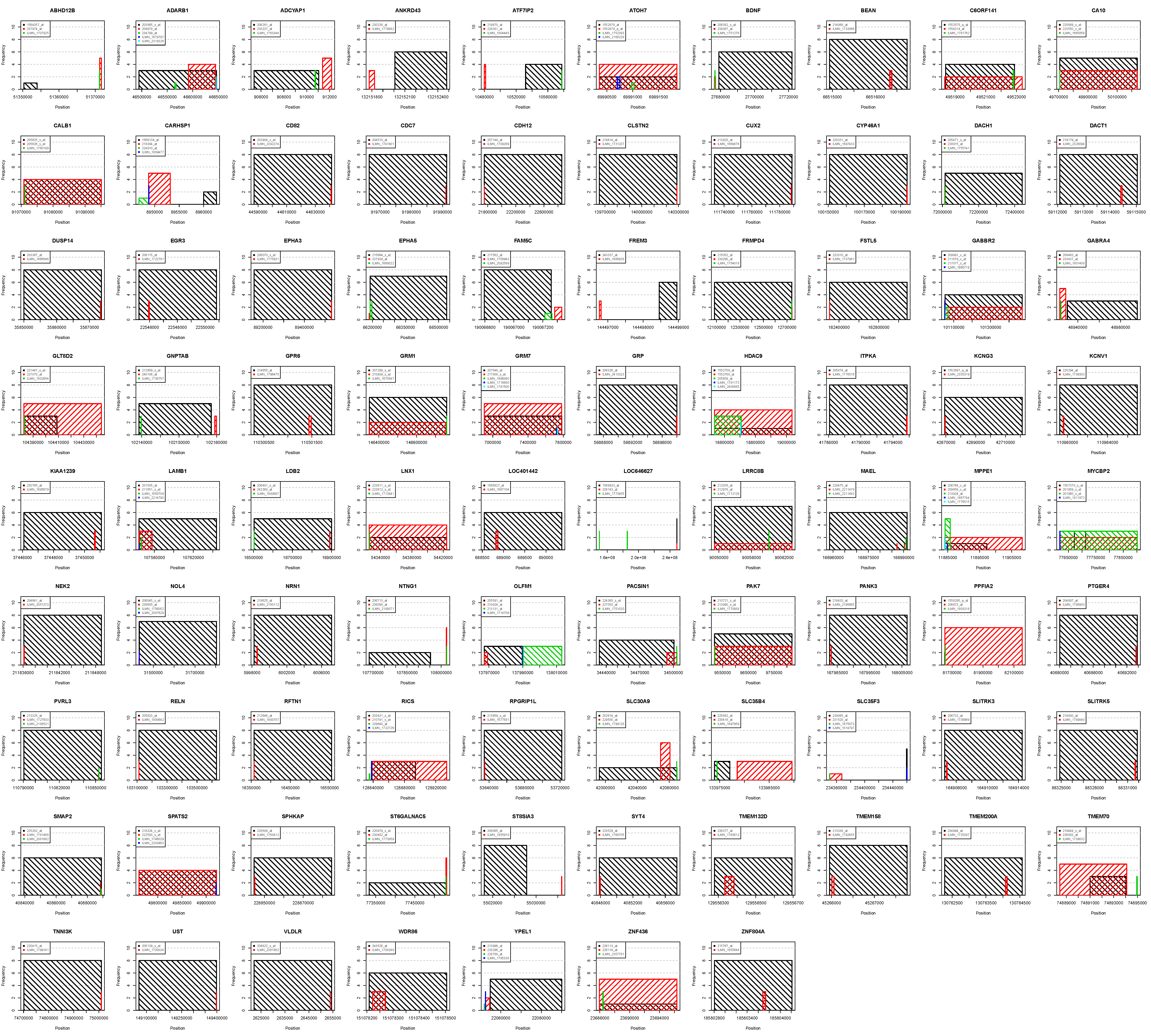

Supplement: Figure S3 — Sequence target overlap between Affymetrix and Illumina array probesets. We have systematically mapped the respective probes that were chosen by our approach and used genes in module #35 to specifically look at overlap in targeted regions. As shown in the individual graphs below, there is overlap in regions for 94% of the genes, indicating that for a few exceptions the same transcript region is used. A histogram represents a chromosomal area of a target sequence in either affymetrix or illumina platform. Wider histogram means the target sequence span over DNA sequence more widely. The height of each histogram shows the number of studies use that specific probe. See main text for additional information. (TIFF) [file pone.0090980.s003.tif]
